# New Molecular Knowledge Towards the Trigemino-Cardiac Reflex as a Cerebral Oxygen-Conserving Reflex

**DOI:** 10.1100/tsw.2010.71

**Published:** 2010-05-04

**Authors:** N. Sandu, T. Spiriev, F. Lemaitre, A. Filis, B. Schaller

**Affiliations:** ^1^ Department of Neurosurgery, University of Paris, France; ^2^ Faculty of Sciences of Sport, University of Rouen, France; ^3^ Department of Neurosurgery, University of Erlangen, Germany

**Keywords:** oxygen-conserving reflex, diving reflex, trigemino-cardiac reflex

## Abstract

The trigemino-cardiac reflex (TCR) represents the most powerful of the autonomous reflexes and is a subphenomenon in the group of the so-called “oxygen-conserving reflexes”. Within seconds after the initiation of such a reflex, there is a powerful and differentiated activation of the sympathetic system with subsequent elevation in regional cerebral blood flow (CBF), with no changes in the cerebral metabolic rate of oxygen (CMRO_2_) or in the cerebral metabolic rate of glucose (CMR_glc_). Such an increase in regional CBF without a change of CMRO_2_ or CMR_glc_ provides the brain with oxygen rapidly and efficiently. Features of the reflex have been discovered during skull base surgery, mediating reflex protection projects via currently undefined pathways from the rostral ventrolateral medulla oblongata to the upper brainstem and/or thalamus, which finally engage a small population of neurons in the cortex. This cortical center appears to be dedicated to transduce a neuronal signal reflexively into cerebral vasodilatation and synchronization of electrocortical activity; a fact that seems to be unique among autonomous reflexes. Sympathetic excitation is mediated by cortical-spinal projection to spinal preganglionic sympathetic neurons, whereas bradycardia is mediated via projections to cardiovagal motor medullary neurons. The integrated reflex response serves to redistribute blood from viscera to the brain in response to a challenge to cerebral metabolism, but seems also to initiate a preconditioning mechanism. Previous studies showed a great variability in the human TCR response, in special to external stimuli and individual factors. The TCR gives, therefore, not only new insights into novel therapeutic options for a range of disorders characterized by neuronal death, but also into the cortical and molecular organization of the brain.

In the last few years, the so-called "oxygen-conserving reflexes" (OCR) [1] have been gaining increasing interest, especially among neurosurgeons and other neuroscientists[2,3,4,5,6,7,8]. This term was coined by the research work of Wolf[2], Andersson and Schagatay[6], and Schaller et al.[9], who studied oxygen consumption in resting human beings. They found that apneic situations with bradycardia were associated with a slightly smaller reduction in arterial oxygen saturation than apneic situations without bradycardia. The OCR is a universal response in animals to oxygen deprivation.

A typical example of these OCR in natural life is the "dive reflex" (DR) observed in diving mammals. It is a protective OCR aimed to keep the body alive during submergence in cold water, preparing itself to sustain life[1,3,10,11]. It is elicited by contact of the face with cold water and involves breath holding, intense peripheral vasoconstriction, bradycardia, decreased ventilation, and increased mean arterial pressure, maintaining the heart and the brain adequately oxygenated at the expense of less hypoxia-sensitive organs[10,12]. In the absence of struggling, the DR can be very profound and can add an enormous margin to the preservation of the brain function called OCR. Many diving mammals, e.g., ducks, smaller penguins, fur seals, and Weddell seals, seem able to metabolize aerobically during diving, as triggered by the DR, at approximately the same (if not greater) rate as they do at the surface[13]. Their enhanced oxygen stores are able to support aerobic metabolism at what would not be considered unusually low levels, for the duration of the dives, although there are probably circulatory readjustments to ensure that the oxygen stores are managed judiciously[13]. For other species, such as the larger penguins, South Georgian shag, and female elephant seals, there is a general consensus that they must either be reducing their aerobic metabolic rate when diving, possibly by way of regional hypothermia, and/or producing ATP, at least partly, by anaerobiosis and metabolizing lactic acid when at the surface[13,14].

Circulation is the proximate regulator of metabolism during aerobic diving and heart rate is the best single indicator of circulatory adjustment[13]. The DR is strong enough to override other seemingly vital reflexes; i.e., it can completely obliterate the tachycardia that accompanies exercise that is usually moderately severe. During voluntary dives, heart rates range from extreme bradycardia to well above resting, reflecting metabolic performance. Reflex cardiorespiratory responses, such as TCR or DR, are modulated by conditioning and habituation, but reflexes predominate during extended dives and during recovery, when gas exchange is maximized[13].

Human beings are not able to hold their breath for as long as diving mammals. This might be due to a less-developed diving response[1]. However, there are many reports of full recovery of near-drowning victims in cold water with immersion times of 30 min or longer. A very illustrative study of the effects of the DR was done with ducks. A duck strangulated with a noose around its neck survived for 4 min in room air. When immersed in water under the same circumstances, the survival time increased to 15 min due to the DR. However, the DR is considered to play a major role in the etiopathogenesis of sudden infant death syndrome (SIDS) or crib death, whose underlying pathological substrates are considered to be mostly congenital in nature and involving the brainstem[15,16]. Another example of OCR in human beings represents the TCR, which was first reported by Schaller et al.[16] during surgery in the cerebellopontine angle (see Fig. 1). It was observed that electrical, mechanical, or chemical manipulation of the trigeminal nerve on its intra- or extracranial course may provoke a drop in mean arterial blood pressure and bradycardia[8,16,17] (see Table 1). Very little is known of these two principal clinical examples of reflexogenic aberrance, and the international literature seems a long way from providing the exact pathophysiology of TCR and DR. It appears, however, that the DR may rather be a subphenomenon and that the TCR is the superordinate principle[2,3,4,5]. Certain cardiovascular mechanisms are present at birth, but are not similar into adulthood. The OCR is, for example, more powerful in the newborn than in the adult. The changing status of developing innervation and regulation of the heart may predispose the neonate to a clinical pathological state such as SIDS. Understanding both these OCR would be of enormous clinical importance in order to resolve major problems, especially during surgery or invasive procedures, but also the dreaded SIDS.

It is generally accepted that the DR and ischemic tolerance involve, at least in part, similar physiological mechanisms[8,12,17]. As regards TCR, it seems to be the higher principle, with the DR being one specific clinical manifestation among others when stimulating the trigeminal nerve. The discovery of those endogenous neuroprotective strategies underlines the clinical importance of TCR. Even though no convincing experimental data exist, TCR may be a specific example of a group of related responses generally defined by Wolf as "OCR"[2]. Within seconds of the initiation of such a reflex, there

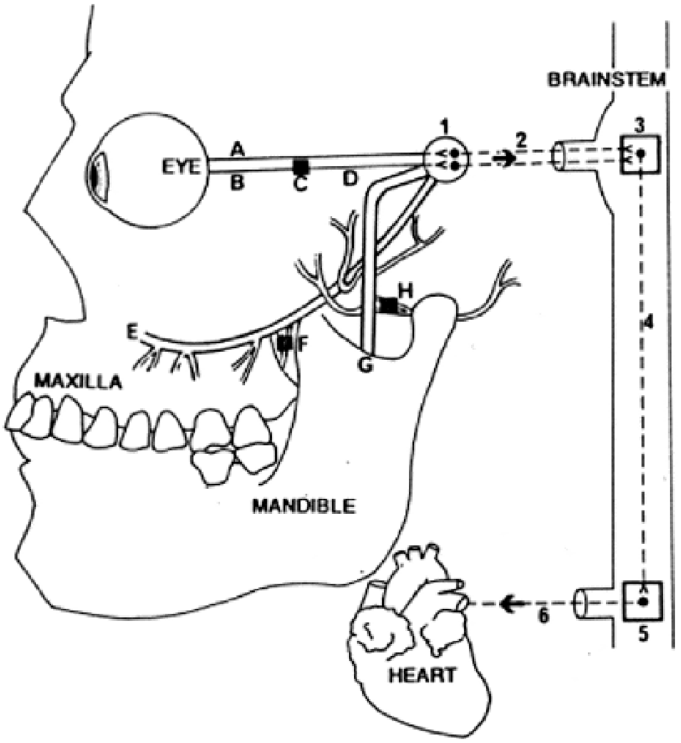

**FIGURE 1.** Drawing illustrating the pathway of the trigemino-cardiac reflex. (Adapted from Lang et al. [1991] *Can. J. Anaesth*. **38,** 757–760 and Schaller et al. [1999] *J. Neurosurg*. **90,** 215–220.) A = long ciliary nerve; B = short ciliary nerve; C = ciliary ganglion; D = ophthalmic nerve; E = maxillary nerve; F = pterygopalatine ganglion; G = mandibular nerve; H = otic ganglion; 1 = gasserian ganglion; 2 = trigeminal nerve; 3 = sensory nucleus of trigeminal nerve; 4 = short internucial fibers; 5 = motor nucleus of vagus nerve; 6 = vagus nerve.

**TABLE 1**
**Some Examples of Afferent Pathways Involved in Trigemino-Cardiac Reflex**

| Sensory Nerve and/or Branches | Trigeminal Division |
|---|---|
| Frontal supratrochlear nerves | V1 |
| Recurrent tentorial branches | V1 |
| Superior alveolar branches | V2 |
| Maxillary nerve branches | V2 |
| Zygomaticotemporal branches | V2 |
| Auricoltemporal nerves | V3 |
| Inferior alveolar nerve | V3 |

Adapted from Schaller et al. [1999] *J. Neurosurg*. **90,** 215–220.

is a powerful and differentiated activation of the sympathetic system[2]. The subsequent elevation in cerebral blood flow (CBF) is neither associated with changes in the cerebral metabolic rate of oxygen ($CMRO_2$) nor with the cerebral metabolic rate of glucose (CMRglc). Hence, it represents a primary cerebral vasodilatation[2]; a state in which the arterial blood pressure seems not to have any influence. However, a temporary reduction in peripheral consumption of oxygen resulting in a slower oxygen uptake from the alveolar space to the blood would temporarily conserve oxygen for the benefit of the central nervous system and the heart, which cannot sustain their metabolism without oxygen.

It has been largely shown that various noxious stimuli may, when applied below the threshold of brain damage, induce tolerance in the brain against a subsequent deleterious stimulus of the same or even another modality; these phenomena are called “ischemic preconditioning” (ICP) and “cross-tolerance”, respectively[16,18]. They probably involve separate systems of neurons of the central nervous system[8]. One of these two systems that mediate reflexive neurogenic protection emanates from oxygen-sensitive sympathoexcitatory reticulospinal neurons of the rostral ventrolateral medulla oblongata. These cells, excited within seconds by a reduction in CBF or $CMRO_2$, initiate the systemic vascular components[19]. They profoundly increase regional CBF without changing $CMRO_2$ or $CMR_{glc}$ and, hence, rapidly and efficiently provide the brain with oxygen[19]. The exact projections are currently undefined. They are thought to project from the rostral ventrolateral medulla oblongata to the upper brainstem and/or thalamus, and finally project to the small population of cortical neurons. These appear to be dedicated to transduce a neuronal signal reflexively into cerebral vasodilatation and synchronization of electrocortical activity[19]. Reticulospinal neurons of the rostral ventrolateral medulla oblongata are “premotor” neurons and, as such, are critical for detecting and initiating the vascular, cardiac, and respiratory responses of the brainstem to hypoxia and ischemia[20]. The systemic response to excitation of rostral ventrolateral medulla oblongata neurons, however, results from activation of a network of effector neurons distributed elsewhere in the central nervous system[20]. Thus, sympathetic excitation is mediated by projections to spinal preganglionic sympathetic neurons, whereas bradycardia is mediated by projections to cardiovagal motor medullary neurons[8,19]. The integrated response serves to redistribute blood from viscera to the brain in response to a challenge to cerebral metabolism[20]. The second mechanism that protects the brain itself from ischemia is represented by the intrinsic neurons of the cerebellar fastigial nucleus and mediates a conditioned central neurogenic neuroprotection. This mechanism is activated by excitation of the intrinsic neurons of the fastigial nucleus and is independent of the first mechanism. These two mechanisms initiate the systemic components of the oxygen-conserving TCR within seconds of excitation[20]. The CBF is significantly increased without changing $CMRO_2$ and, thus, the brain is rapidly provided with oxygen. These mechanisms described above need a pre-exposure that can be seen clinically by a repetitive stimulation of the TCR; for example, during an operation[16]. That the brain may have neuronal systems dedicated to protecting itself from ischemic damage at first appears to be a new concept. However, upon reflection, this is not surprising given that there exists a naturalistic behavior characterized by very low levels of regional CBF, such as diving or hibernation[12]. The exact mechanisms of neurogenic neuroprotection are unknown, but such neuroprotective adaptation may be part of preconditioning strategies[20]. Probably, these reflexes, like the TCR, may prevent other brain insults as well, which therefore remain unrecognized.

Activation of the trigeminal pathway often evokes action potentials in cardioinhibitory vagal neurons (CVNs). Application of the serotonin (5-HT) reuptake inhibitor citalopram significantly enhances these responses. Consistent with the hypothesis, this pathway is endogenously modulated by the 5-HT receptors: 5-HT1A receptor antagonist inhibits, whereas the 5-HT2A/C receptor antagonist facilitates the excitatory neurotransmission to CVNs[21,22]. Contrarily, the 5-HT1A receptor agonist increases, whereas the 5-HT2 receptor agonist inhibits this TCR pathway[22]. These results indicate that stimulation of trigeminal fibers evokes a powerful excitatory and polysynaptic pathway to CVNs, and this pathway is endogenously modulated and differentially enhanced and depressed by 5-HT1A and 5-HT2 receptors, respectively[20]. However, 5-HT2 receptors differentially modulate excitatory neurotransmission to CVNs during and after hypoxia[23]. Activation of 5-HT2 receptors functions to maintain excitatory neurotransmission to CVNs during hypoxia, likely via presynaptic facilitation of 5-HT3 receptor–mediated neurotransmission to CVNs[23]. However, activation of 5-HT2 receptors diminishes the subsequent inspiratory-related excitatory neurotransmission to CVNs that is recruited during the recovery from hypoxia, likely exerting an inhibitory action on inspiratory-related purinergic signaling[23]. Such synapses between the autonomic postganglionic neuron and effector tissue, the neuroeffector junction, differ greatly from neuron-to-neuron synapses[24]. The postganglionic fibers in the autonomous nervous system do not terminate in a single swelling like the synaptic knob, nor do they synapse directly with the cells of a tissue[24]. Instead, where the axons of these fibers enter a given tissue, they contain multiple swellings called varicosities[24]. When the neuron is stimulated, these varicosities release neurotransmitters along a significant length of the axon and, therefore, over a large surface area of the effector tissue[24]. The neurotransmitter diffuses through the interstitial fluid to wherever its receptors are located in the tissue[24]. This diffuse release of the neurotransmitter affects many tissue cells simultaneously[24]. Furthermore, cardiac muscles have gap junctions between cells[24]. These specialized intercellular communications allow for the spread of electrical activity from one cell to the next[24]. As a result, the discharge of a single autonomic nerve fiber to an effector tissue may alter the activity of the entire tissue[24].

Accordingly, it can be suggested that the TCR represents a "physiological" entity rather than a pathological one. Better and more detailed knowledge of the cascades, transmitters, and molecules engaged in such endogenous protection may provide new insights into novel therapeutic options for a range of disorders characterized by neuronal death and into the cortical organization of the brain. Hypoxic or anoxic tolerance is found ubiquitously in nature, especially in diving and hibernating species[24,25,26]. A common feature in most anoxic-tolerant species or during hibernation is a pronounced metabolic depression[27]. For example, it is now well accepted that during diving, turtle brains undergo metabolic depression, which is characterized by a depression in electrical activity[28,29].

One question that arises in the field of IPC is whether it induces metabolic depression in a similar manner as that observed in diving vertebrate species or during hibernation. Up to now, there are no studies that have directly demonstrated that electrical activity in the mammalian brain is particularly depressed after IPC. However, the evidence points towards that fact. It is well established that inhibitory pathways are enhanced after IPC. For example, several groups have demonstrated that glutamate release during ischemia was ameliorated by several forms of preconditioning[29,30,31,32], and down-regulation of the excitatory receptors alpha-amino-3-hydroxy-5-methyl-4-isoxazolepropionic acid (AMPA)[33] and N-methyl-D-aspartic acid (NMDA)[34] also occurred. In contrast to glutamate, increases in gamma-aminobutyric acid (GABA) release were observed after IPC[31,32]. These results also suggest that IPC, as in diving species, promotes a metabolic rate down-regulation in the brain, thus reducing energy-consuming pathways.

In the DR, however, adapted species must be metabolically prepared to respond to a potential hypoxic insult. It is possible that activation of the DR by shifting blood flow to the brain provides additional time that allows the brain to prepare for the eventuality that anoxia ensues, by activating signaling pathways similar to those observed as triggers of IPC.

Two good candidates to trigger a neuroprotective cascade during the DR and IPC are adenosine and the activation of the ATP-sensitive potassium channel. Several studies have demonstrated the role of the adenosine A1 receptor in both anoxia tolerance in diving species and in IPC[33,34,35,36, 37,38,39,40,41,42]. Activation of the K_ATP channel likely plays a role in at least some of the mechanisms of IPC[34,43]. However, the precise K_ATP channel involved remains undefined. Recently, two ATP-sensitive potassium channels have been described. One of these channels resides in the plasma membrane; the other resides in the mitochondrial inner membrane. The mtK_ATP has been suggested to be the key channel involved in IPC[44,45]. It has been suggested that opening of the mtK_ATP channel may depolarize mitochondrial membrane potential, promoting an increase in the electron transport chain rate and thus increasing ATP production[46]. These two triggers are the logical result of the oxygen-sensing mechanism, because they are both linked to ATP levels. Once they are activated, a number of signaling pathways ensue that orchestrate the anoxic-/ischemic-tolerant phenotype. For a more in-depth description of some of these signaling pathways and genes expressed after IPC see Gidday[18].

Further improvement in knowledge may be assigned by state-of-the-art imaging methods in the next few years: first in animal models, then in human beings, and finally during operations. Recent clinical studies suggest the existence of such an endogenous neuronal protective effect in the human brain[47,48,49] and represent a rational basis for the development of neuroprotective drugs. However, previous studies showed a great variability in the human TCR response, in special to external stimuli and individual factors. We speculate that the oxygen-conserving potential, if any, of TCR responses in humans might not be demonstrable in resting humans, but becomes evident only during stress (such as diving or operations), when oxygen requirements are increased. We additionally hypothesize that the subjects with the most marked responses to apnea also would show the highest degree of oxygen conservation, manifested as the slowest rate of $Sa_{O2}$ decline. Given the potential hazard of inducing ischemic tolerance by the TCR in human beings, a trial may not be advisable, and proof of testing of agents that safely mimic the effects of the TCR may be required.
